# Granulocyte Colony-Stimulating Factor Receptor Mutations in Myeloid Malignancy

**DOI:** 10.3389/fonc.2014.00093

**Published:** 2014-05-01

**Authors:** Clifford Liongue, Alister Curtis Ward

**Affiliations:** ^1^School of Medicine, Deakin University, Geelong, VIC, Australia; ^2^Strategic Research Centre in Molecular and Medical Research, Deakin University, Geelong, VIC, Australia

**Keywords:** G-CSF, G-CSFR, *CSF3R*, AML, SCN, CNL, MDS

## Abstract

Granulocyte colony-stimulating factor is a cytokine able to stimulate both myelopoiesis and hematopoietic stem cell mobilization, which has seen it used extensively in the clinic to aid hematopoietic recovery. It acts specifically via the homodimeric granulocyte colony-stimulating factor receptor (G-CSFR), which is principally expressed on the surface of myeloid and hematopoietic progenitor cells. A number of pathogenic mutations have now been identified in *CSF3R*, the gene encoding G-CSFR. These fall into distinct classes, each of which is associated with a particular spectrum of myeloid disorders, including malignancy. This review details the various *CSF3R* mutations, their mechanisms of action, and contribution to disease, as well as discussing the clinical implications of such mutations.

## G-CSF and Its Receptor

Granulocyte colony-stimulating factor (G-CSF, also called CSF3) augments the production and function of neutrophilic granulocytes, which play an essential role combatting infection, especially those of a bacterial or fungal nature (1–5). G-CSF acts to mobilize hematopoietic precursor cells and stimulate the proliferation and differentiation of myeloid cells, particularly along the neutrophilic lineage, as well as activate various functions of mature neutrophils (6). These properties have seen G-CSF widely used in the treatment of neutropenic conditions, including severe congenital neutropenia (SCN) (7–9), and those associated with chemotherapy (10–12). G-CSF has also been extensively used for harvesting of HSCs from the peripheral blood, thereby overcoming the requirement for bone marrow transplantations in many instances (13, 14).

The biological actions of G-CSF are mediated via docking to a homomeric receptor found on the surface of target cells, granulocyte colony-stimulating factor receptor (G-CSFR) (also called CSF3R) (15). The G-CSFR is a member of the hematopoietin receptor superfamily, which has no intrinsic tyrosine kinase activity but upon ligand-binding undergoes conformational changes to stimulate multiple tyrosine kinases associated with its cytoplasmic domain. These include Janus kinases (JAKs), especially JAK1 and JAK2 (16–19), members of the SRC kinase family, especially LYN and HCK (20–22), as well as SYK (20) and TNK (23). Important pathways activated downstream include the signal transducer and activator of transcription (STAT) proteins, particularly STAT3 and STAT5 (17, 18, 24, 25), the phosphatidyl inositol 3-kinase (PI3-K)–AKT pathway (21, 26, 27), and the RAS–MAPK pathway (28–30). Signaling via the G-CSFR is tightly regulated, including by members of the SOCS family, especially SOCS3 and CISH (31, 32), as well as the tyrosine phosphatases SHP-1 (26, 33) and SHP-2 (34, 35).

## Role of G-CSFR Mutations in Myeloid Disorders

**Figure 1:**
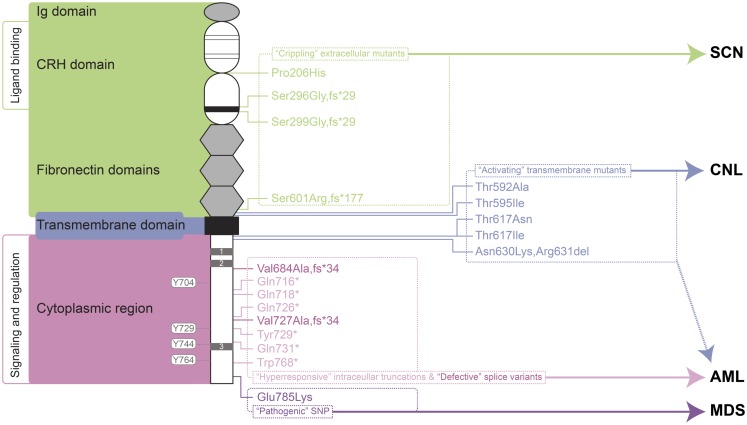
**Granulocyte colony-stimulating factor receptor perturbations in disease**. Schematic representation of the mature G-CSFR (RefSeq NP_000751.3), showing important subdomains and residues conserved among members of the hematopoietin receptor superfamily, including the N-terminal Ig domain, four conserved cysteines (thin line), and WSXWS motif (thick line) within the CRH domain, fibronectin, and transmembrane domains as well as Box 1–3 (gray rectangles) and important tyrosine residues within the cytoplasmic region. The relative positions of various classes of mutation are indicated on the right along with the respective clinical manifestations of these and other G-CSFR perturbations. Abbreviations: Ig, immunoglobulin-like; CRH, cytokine receptor homology; SCN, severe congenital neutropenia; CNL, chronic neutrophilic leukemia; MDS, myelodysplastic syndrome; AML, acute myeloid leukemia.

A large number of mutations in the gene encoding the G-CSFR, designated *CSF3R*, have now been described. These mutations can be placed into a number of distinct classes that relate to the type of mutation and their biological and clinical consequences (Figure 1). Mostly these relate to perturbations of the myeloid lineage, including SCN, Myelodysplastic syndrome (MDS), acute myeloid leukemia (AML), and chronic neutrophilic leukemia (CNL). To avoid potential confusion over mutation nomenclature, this review provides residue numbers relative to those of the mature G-CSFR in the format suggested by the Human Genome Variation Society, but with the alternate numbering that includes the cleaved signal sequence given in parenthesis in each case, since these have also been used in the literature.

### “Crippling” extracellular mutants

One class of mutations has been identified affecting the extracellular domain of the G-CSFR in patients with SCN (36–38) or chronic idiopathic neutropenia (CIN) (39). These mutations have in common the property of not only being defective themselves, but also activating in a dominant-negative manner to cripple co-expressed wild-type receptors (36–38). The first of these mutations described was a germline p.Pro206His (p.Pro229His) change that disrupted a conserved di-proline “hinge” motif located between two halves of the ligand-binding cytokine receptor homology (CRH) domain. This disrupted the normal architecture of the ligand/receptor complex, with severe consequences for G-CSF-mediated signal transduction and cellular responses (36). Two other mutants represent deletions of the *CSF3R* gene and concomitant alterations in reading frame that yield G-CSFR proteins consisting of extracellular regions truncated at the WSXWS motif followed by a novel sequence and a premature stop: the somatic p.Ser296Gly,fs*29 (p.Ser319Gly,fs*29) mutation (38) and the germline p.Ser299Gly,fs*29 (p.Ser322Gly,fs*29) (37). Finally, the CIN-associated p.Ser601Arg,fs*177 (p.Ser624Arg,fs*177) mutation involved a frameshift that truncates the receptor after the fibronectin domains (39). While not directly promoting malignancy, the neutropenic conditions that this class of mutation produces are likely to create susceptibility to other changes that can. Indeed, one SCN patient with this type of mutation subsequently acquired additional truncating mutations in the G-CSFR (40), while the CIN patient went on to develop acute myeloid/natural killer cell leukemia, although whether the *CSF3R* mutation played a role in the latter was not determined (39).

### “Activating” transmembrane mutants

Another class of *CSF3R* mutations affects the transmembrane domain and adjacent residues of the encoded receptor. This class of mutations appears to act by stabilizing transmembrane helix–helix interactions in the absence of ligand, creating an active dimeric configuration that leads to constitutive (and enhanced) activation (41). These are analogous to the activating mutations found in the thrombopoietin receptor, c-MPL, which are associated with hereditary or acquired thrombocythemia (42, 43), or those in the βc chain of the heterodimeric IL-3R family identified *in vitro* (44, 45).

The p.Thr595Ile (p.Thr618Ile) mutation was initially described as a late somatic mutation in the development of AML in an SCN patient already bearing an alternate G-CSFR mutation (46). However, p.Thr595Ile has subsequently been identified as a common mutation in CNL (23, 47), with the adjacent p.Thr592Ala (p.Thr615Ala) mutation alternatively found in other cases of CNL (23). The p.Thr595Ile mutation is also less commonly observed in atypical chronic myelogenous leukemia (aCML) (23), chronic myelomonocytic leukemia (CMML) (48), *de novo* AML (23, 48, 49), as well as in cases of early T-cell precursor acute lymphoblastic leukemia (ETP-ALL) (23). G-CSFR forms containing either the p.Thr595Ile or p.Thr592Ala mutation supported G-CSF-independent growth of Ba/F3 cells, although growth was similar to wild-type receptor at high G-CSF concentrations (48). Bone marrow transduced with the p.Thr595Ile mutant also resulted in G-CSF-independent growth (46), which could be replicated by a p.Thr595Val mutant, suggesting the change to a hydrophobic amino acid was sufficient (49). Ba/F3 cells expressing the p.Thr595Ile mutant showed constitutive activation of JAK2, SRC, TNK, STAT3, and STAT5 (23, 48), but not AKT and ERK, as well as enhanced ROS production (48). Signaling from the mutant was found to be sensitive to various JAK kinase inhibitors, including ruxolinitib and tofacitinib (23, 48), with some evidence of clinical efficacy (23), but not to dasatinib that targets a number of tyrosine kinases, including SRC and TNK (23, 48).

The p.Thr617Asn (p.Thr640Asn) mutation was first identified in a single case of AML (50). Further studies identified this – and the alternate p.Thr617Ile (p.Thr640Ile) – as rare, somatic mutations in *de novo* AML (49, 51). However, a germline p.Thr617Asn mutation was also identified as the cause of autosomal dominant hereditary neutrophilia, where it showed complete penetrance (52). Interestingly, one of the affected individuals progressed to a myelodysplastic syndrome type disease (52), further implicating this mutation as predisposing toward myeloid malignancy. In addition to neutrophilia, patients harboring p.Thr617Asn possessed increased numbers of CD34+ cells, which were able to proliferate and terminally differentiate in the absence of G-CSF, and induce a myeloproliferative (MPD)-like disorder in mice. Patient CD34+ cells showed constitutive phosphorylation of JAK2, STAT3, STAT5, and ERK, which were hyperactivated by G-CSF compared to wild-type cells (52). Lineage-negative bone marrow cells retrovirally transduced with the p.Thr617Asn mutant G-CSFR caused neutrophilia when transplanted into irradiated mice (52). The p.Thr617Asn mutation also supported factor-independent growth and survival in Ba/F3 cells, with weak constitutive phosphorylation of the receptor, JAK2, STAT3, and ERK, and also enabled transduced CD34+ cells to undergo myeloid differentiation in the absence of G-CSF (51).

Finally, an in-frame three nucleotide deletion has been identified in MDS that replaces two amino acids with an alternate residue, p.Asn630Lys,Arg631del (p.Asn653Lys,Arg654del). This mutation resulted in prolonged signaling following ligand stimulation (53).

### “Hyperresponsive” intracellular truncations

By far the most studied clinical abnormalities of the *CSF3R* gene are a series of acquired nonsense mutations identified in a subset of SCN patients with a propensity to progress to leukemia. These somatic mutations typically affect a single allele to truncate between 82 and 98 amino acids from the carboxyl-terminus of the receptor, such as p.Gln718* (p.Gln741*) and pGln731* (p.Gln754*) (54, 55). These truncated receptors show normal affinity for G-CSF (56), but mediate heightened growth and diminished maturation in response to ligand, acting dominantly over wild-type receptors (54). Truncated G-CSFRs are not the primary cause of SCN, although they may exacerbate it to a modest extent (57–60). However, it is clear that SCN patients carrying truncating G-CSFR mutations show a particularly strong predisposition to both MDS and AML (61, 62). Indeed in SCN patients progressing to AML, the most common mutations identified are in *CSF3R* (82%), followed by *RAS* (~50%) and monosomy 7 (63), and when *CSF3R* mutations are present, 100% of blasts carry the mutation (54, 63). However, since mutations are not always seen in AML and can spontaneously disappear (64), progression to leukemia is not inevitable.

A mouse line carrying a truncated G-CSFR “knock-in” allele (57) or one transgenically expressing a truncated human G-CSFR (58) exhibited mild neutropenia, with an increased percentage of immature myeloid cells that were defective in maturation *ex vivo* (58, 65). An alternate mouse line with a targeted receptor truncation displayed normal neutrophil numbers, although the truncated form of the receptor was significantly overexpressed relative to the wild-type (59). However, all three studies revealed a hyper-responsiveness to G-CSF, with exogenous G-CSF producing elevated numbers of neutrophils compared to wild-type mice (57–59), due to increased myeloid progenitor proliferation (58, 65). Another study confirmed that G-CSFR truncations conferred a strong clonal HSC advantage that was also dependent on exogenous G-CSF (66), providing insight into how these mutants may contribute to their frequent progression to MDS/AML. Notably, expression of the truncated receptor in mice was not by itself leukemogenic, since no spontaneous leukemia has been reported in mice hetero- or homozygous for the mutation (57, 59). However, the truncated G-CSFR was found to co-operate with PML–RARa to induce AML in mice, where it decreased latency in a G-CSF-dependent manner, leading to higher blast counts and increased myelosuppression (67).

Investigation into the molecular mechanisms of G-CSFR signal transduction has helped to explain the dominant hyperproliferative function of truncated G-CSFRs. These mutant receptors exhibit higher and more sustained activation in comparison to wild-type receptors, with a heavily reduced “off-rate” (65, 68, 69). This is partly a result of impaired internalization due to the combined loss of a conserved di-leucine containing motif in Box 3 (69, 70), and a less well-defined motif spanning residues 756–769 (34). However, direct negative regulation is also blunted, due to the loss of recruitment sites on the truncated receptors, including those for the receptor-associated tyrosine phosphatases SHP-1 (at an undefined site in the C-terminus) (71) and SHP-2 (at Y724) (34), and two members of the SOCS family, CISH (at Y729 and Y744) (32) and SOCS3 (at Y729) (34), the latter exacerbated by decreased *SOCS3* transcription as a result of reduced STAT3 activation by truncated receptors (34).

Cells expressing truncated G-CSFR receptors are also hypersensitive to ligand (54, 70). This is associated with an altered dose–response of STAT3:STAT5 activation, the ratio of which is drastically reduced at low concentrations of G-CSF (24). Since STAT5 contributes to G-CSF proliferative responses (72), while STAT3 is inhibitory (73–75), the reduced STAT3:STAT5 ratio may shift the balance toward proliferation, explaining the G-CSF hypersensitivity (54, 56).

Granulocyte colony-stimulating factor receptor truncation impacts on the length and magnitude of receptor activation, and particularly of STAT5 (69–71), pathways downstream of PI3-K, such as AKT (27, 76), as well as SRC (23). Dominant-negative STAT5 has been shown to inhibit the hyperproliferative function of truncated G-CSFRs *in vitro* (77), while the absence of STAT5 abrogated the clonal HSC advantage conferred by these receptors *in vivo* (66). Other pathways also contribute to proliferation and survival, including PI3-K, MAPK, and STAT3 (76–78). Interestingly, receptor truncations are sensitive to the multi-kinase inhibitor dasatinib, but not to JAK inhibitors (23), suggesting an intrinsic difference in comparison to the activating transmembrane mutants. Truncated receptors have also been shown to increase ROS production (79), potentially creating genotoxic stress to facilitate addition mutations in cells expressing these receptors.

### Defective splice variants

A presumably somatic single base change in *CSF3R* adjacent to a cryptic splice-donor site has been identified in blasts of a *de novo* AML patient. This resulted in high expression of an alternate splice variant that generated a G-CSFR protein in which the C-terminal 130 amino acids are replaced with a different 34 amino acids from an alternate reading frame, p.Val684Ala,fs*34 (p.Val707Ala,fs*34) (80). The primary AML blast cells of this patient failed to respond to G-CSF in proliferation assays *in vitro*, despite responsiveness to IL-3 or GM-CSF being maintained. This variant was unable to transduce either proliferation or maturation signals in murine cell systems. By corollary, AML cells show a tendency for significantly increased levels of a normally minor *CSF3R* transcript, class IV (81), which encodes a similar G-CSFR protein in which the C-terminal 87 amino acids are replaced with the same alternate 34 amino acids, p.Val727Ala,fs*34 (p.Val750Ala,fs*34). The authors argue that the altered balance of class IV to normal (class I) receptors might contribute to AML, due to the ability of the class IV receptor to block maturation.

### Pathogenic SNP

A *CSF3R* SNP that is present in ~6% of the population leads to a p.Glu785Lys (p.Glu808Lys) amino acid substitution in the intracellular region of the G-CSF, which predisposes individuals to high-risk MDS (82). Interestingly, blasts from an individual who developed AML following high-risk MDS were found to be homozygous for this allele (83), providing further evidence of the potential pathogenicity of this SNP. Although the mechanism of action remains unknown, the variant receptor appears functional, but can act in a dominant-negative manner to reduce colony formation compared to the wild-type receptor (82, 83).

## Conclusion

**Figure 2:**
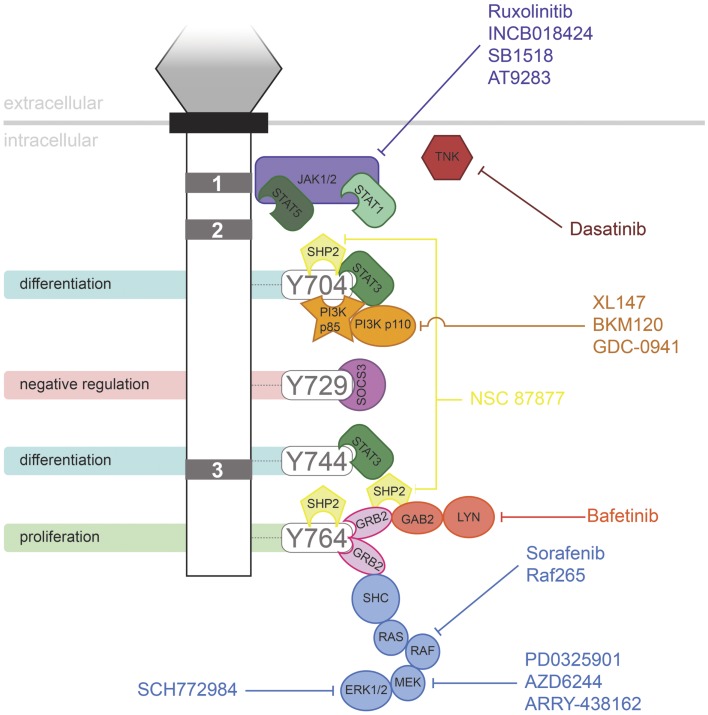
**Inhibitors of signaling pathways downstream of G-CSFR**. Schematic representation of the intracellular domain of G-CSFR, showing the important Box 1–3 sequences (gray rectangles), as well as the tyrosine residues that serve important docking sites for the downstream signaling proteins indicated. Known inhibitors of these are shown.

Granulocyte colony-stimulating factor has proven to be an effective therapy in a range of life-threatening conditions or to aid in the recovery of medical treatments, such as in the treatment of neutropenia following chemotherapy. However, the evidence suggests that G-CSFR mutations contribute to several disorders, including in settings where G-CSF may be used therapeutically. It has been suggested that use of G-CSF in SCN may allow the selective expansion of clones containing truncating *CSF3R* mutations. However, the available data are complicated, making conclusions difficult. One study reported no significant relationship between age of MDS/AML onset and G-CSF dose or duration of therapy (63). However, another study suggested that the risk of leukemia in SCN patients increased with the degree of G-CSF therapy (84). However, higher doses may also reflect a more severe underlying disease with a higher propensity to MDS/AML. In addition, SCN patients developed AML prior to the advent of G-CSF therapy. In line with this, one SCN patient progressed to CMML in the absence of G-CSF treatment, but expressed a truncated G-CSFR (85). Thus it is possible that the mutant receptor form may have a selective advantage in the absence of treatment, perhaps due to the elevated G-CSF levels seen in SCN patients as a result of their neutropenia (63). However, G-CSF therapy is not a factor in other classes of *CSF3R* mutation, such as those leading to CNL. A number of pharmacologic agents are now available that target signaling pathways downstream of the G-CSFR (Figure 2), providing hope for effective treatment strategies for patients harboring G-CSFR mutations. Indeed, recent studies have begun to elucidate how these might specifically combat the aberrant signaling elicited by “activating” and “hyperresponsive” G-CSFR mutations (23, 48).

## Conflict of Interest Statement

The authors declare that the research was conducted in the absence of any commercial or financial relationships that could be construed as a potential conflict of interest.
